# Local governance of immigrant incorporation: how city-based organizational fields shape the cases of undocumented youth in New York City and Paris

**DOI:** 10.1186/s40878-018-0097-z

**Published:** 2018-11-05

**Authors:** Stephen P. Ruszczyk

**Affiliations:** Montclair State University, One Normal Ave, 313 Dickson Hall, Montclair, NJ 07024 USA

**Keywords:** Undocumented youth, Immigrant incorporation, Organizational field, Immigration, Civil society

## Abstract

City-based organizations and governments play an important role in incorporating undocumented immigrant youth. This article investigates how localities socio-politically incorporate these immigrants by examining the governance constellations and institutional logics of the organizational field that manages undocumented youth. Comparing sets of municipal and civil society organizations in different national settings, I use the two cases of New York City and Paris to ask how the ‘city-based organizational field of immigrant incorporation’ shapes citizenship experiences of undocumented youth. Data come from multi-level longitudinal ethnography over 8 years with two dozen undocumented youth and with organizations in each city as well as interviews with immigrant organization staff and other governance actors in New York and Paris. Organizational field dynamics in Paris provide a stronger possibility of citizenship and rights acquisition, evidence of socio-political incorporation. In contrast, New York’s robust and flexible labor market and ethnic and immigrant legitimacy offer its undocumented youth a marginalized socio-political incorporation. These findings support practice-based understandings of local governance of incorporation of undocumented youth.

## Introduction

Nation-states remain the leading authorities that provide rights to immigrants, shaping labor market access and citizenship acquisition. Within countries, however, cities have also been able to shape the acquisition and practice of rights. For this reason, scholars have increasingly used a multi-level governance lens to represent contested divisions in powers between nation-states, regions, and localities (Cortez, 2008; Dekker, Emilsson, Krieger, & Scholten, 2015; Maas, 2013). Cities manage integration matters, for example by creating inclusionary practices towards immigrants (De Graauw, 2016; Garbaye, 2005; OECD, 2006). Cities have governments, employers, and organizations that may be receptive to immigrants and sensitive in particular to undocumented youth and workers. How do these actors shape citizenship outcomes for undocumented youth?

If we are looking to explain cities’ ability to shape the lives of undocumented youths, large cities such as Paris and New York, with substantial civil societies, would be expected to be best-case scenarios. The embeddedness of each within a national system of government would be expected to shape its influence over immigration practices. Amongst industrialized countries, for example, the US has a weak social welfare system, a loosely-regulated labor market, and a federal government. One would expect a city like New York to exert much authority over the acquisition and practice of rights. Conversely, given the stronger social welfare system and centralized government in France, one would expect that undocumented youths in Paris would not be able to legalize: the costs to the state are much higher than in the US and the state has more control. Surprisingly, undocumented youths are more likely to legalize and gain the associated rights in Paris than in New York. The local interplay of civil society and government explains this unanticipated effect of local context.

Given the salience of legal status to social, economic, and political opportunities, undocumented youth are particularly sensitive to local policies and practices. Local governments’ role in incorporation can vary considerably (Garbaye, 2005; Penninx, Caponio, Garcés-Mascareñas, Matusz Protasiewicz, & Schwarz, 2014; Varsanyi, 2010). By contesting or cooperating with policy, civil society also shapes local geographies of immigrant ‘illegality,’ however (Ruszczyk, 2018). How such context matters for the incorporation of undocumented youth (i.e. local immigrant ‘illegality’) has been much less explored. Engaging concepts from organizational studies, politics, geography, and sociology, this article asks how localities socio-politically incorporate these immigrants by examining the organizational field that manages undocumented youth.

Drawing on longitudinal data collection from two multi-level case studies with undocumented youth and organizations in New York City and Paris, I argue the interplay of state, market, and civil society actors within the local organizational field, more than formal political representation, shapes qualitatively different experiences of citizenship. Two aspects of this local governance of immigrants, governance constellations and institutional logics, shed light on how citizenship outcomes for undocumented youth differ from national models. I present a brief background on the cases of Paris and New York. The findings describe the institutionalized relationships between government and civil society, with a focus on how civil society shapes transaction costs for the government, and the resulting production of citizenship. The socioeconomic incorporation follows what might be expected from national models (e.g. looser labor market regulation in the US facilitates labor participation). In contrast, the New York organizational field produces exclusion along a socio-political axis for undocumented youth while its counterpart in Paris results in undocumented youth socio-political incorporation albeit qua ethnic minorities.

### Organizational field, citizenship, and local incorporation

This analysis engages governance and citizenship to examine obverse facets of a similar social process, immigrant incorporation. The complexities of governing migration have given rise to local initiatives, public-private partnerships, and new relationships between international, national, regional, and local actors. Many actors, those that activate citizenship from above and those that mobilize participation from below, shape how different immigrant groups integrate into the local social and political fabric. Governance, which recognizes these multiple actors, refers to “the rules of collective decision-making in settings where there are a plurality of actors or organizations” without a formal control system (Chhotray & Stoker, 2010, p. 3). An organizational field operationalizes governance within a specific network or space; here, that which structures the social and political incorporation of undocumented youth. An examination of the social and political opportunities of undocumented youth in Paris and New York exposes how the dynamics of the organizational field shape these expressions of citizenship.

Studies of the organizational field began when scholars redefined the ‘external environment’ of an organization to be the dynamics of other organizations that interact and work on similar services or goods (Dimaggio & Powell, 1983; Scott, 2013). The field emerges as actors interact and develop information flows, develop positions of power and coalition, and are aware of their common endeavor (DiMaggio & Powell, 1983, p. 148). This shift to studying the organizational field highlights how much such field-based dynamics shape how social life unfolds by specifying the representation of proximate context, showing structures to be contingent on institutionalized relationships, and clarifying the constraints and agency that manifest within the structured field.

By specifying how social movements become institutionalized (Zietsma & Lawrence, 2010; De Bakker, den Hond, King, & Weber, 2013), organizational field analysis can provide new insights of the discursive, cognitive, and political mechanisms of cooperation and contestation. In reaction to the heightened enforcement of border entry and of legal status in the interior of both France and the US, social movements for the inclusion of undocumented youth have used the local level to contest such restriction and, in so doing, have re-positioned the city-based organizational field (COFI) of New York and Paris. The Dreamer movement in the US and the Jeunes sans-papiers movement in France have leveraged the universal inclusion of minors in schools to call into question their exclusion at adulthood. These movements, each of which began in earnest in the early 2000s, frame undocumented youths as having grown into a native identity with citizen subjectivities (Nicholls, 2013). While they lack formal political participation, access to civil rights gives them the right to participate in most informal means of participation. These movements have been successful in vocalizing claims by and on behalf of undocumented residents. Depending on the ability of civil society to change transaction costs of managing undocumented youth (North, 1990), such claims take hold within the organizational field, and examination of COFI can show *how* such support shifts citizenship possibilities for undocumented youth.

This framework focuses on the cooperative and contentious relations *between* migrant organizations and local governments and between organizations themselves in a way that recognizes the power inequalities that constrain the agency of migrant organizations (Nicholls, 2005). Resources (e.g. financial, personnel, space), presence (i.e. recognition and legitimacy from other actors), and weight (i.e. ability to collaborate and advance interests) influence how organizations are positioned in a given policy area (Ramakrishnan & Bloemraad, 2008). As relations between local governments, organizations, and market actors institutionalize around question of undocumented youths, we recognize those relational elements in the organizational field. These dynamics shape possibilities for immigrant social life, from education to work, community life, and more. Two features of COFI best capture the way it works: its governance constellation and institutional logic(s).

*Governance constellations* represent the structural relations between differently- positioned actors that affect (here) incorporation and respond to field-level contingencies (e.g. national policy or demographic shifts) (Penninx et al., 2014). The makeup of these constellations has clear impact on the lived experiences of undocumented residents. For example, during the implementation of the federal Deferred Action for Childhood Arrivals (DACA) program, which allowed temporary protection for qualified undocumented youth,^1^ places with more immigrant organizations had higher uptakes of applicants (Wong et al., 2013). Local governments may partner with businesses, unions, and civil society not only to provide support services but also to regulate residents without legal status. For example, the French government has used unions in Paris to funnel and limit regularization claims by undocumented workers (Barron, Bory, Chauvin, Jounin, & Tourette, 2011).

Governance constellations give us a map of actors. *Institutional logics* tell us how they act; it is the culture of the organizational field. Discursive and procedural norms reflect the logic of the field. Institutional logics feature legitimate, shared understandings but also allow for a dynamic agency: “A field’s institutional logic provides the organizing principles and practice guidelines for field participants – individually and collectively. Actors in an organizational field ‘both create (produce) and enact (reproduce) the logics of a field’” (Scott, Ruef, Caronna, & Mendel, 2000, p. 172). Organizations, whether public or private, need to be perceived as legitimate in order to survive (DiMaggio & Powell, 1983). Community-based organizations, for example, are subject to certain external norms (e.g. grant writing, professionalization) in order to obtain funding. As conditions change, conflict, cooperation or coercion may push organizational institutional logics to blend, shrink in scope or migrate across fields (Thornton, Ocasio, & Lounsbury, 2012).

Immigration logics differ at the national and local levels (Jørgensen, 2012). We also see contrasts when local government, with its institutionalized logics on immigration, interacts with civil society, with *its* institutionalized logics. Governmental rules and access to financial resources can harmonize or clash with the organizational representation of immigrants depending on the institutional logic; either exchange may result in positive or negative outcomes for immigrant integration. Given that governments and non-profit organizations now regularly cooperate to carry out the delivery of social services, with different organizational outcomes (Wells, 2004), the institutional logics of government and organizations shift and can form a meta-institutional logic at the local, rather than organizational, level. Asymmetrical actors negotiate this interactive process, resulting in a more confrontational clash of organizational institutional logics in Paris and a more unified one in New York (Greenwood, Díaz, Li, & Lorente, 2009). New York’s COFI unifies institutions (e.g. schools, employers, religious institutions) that act on immigrants operate under different logics to form a general logic of ethnic-based inclusion for workers (Treitler, 2013). The state-heavy COFI in Paris legitimizes human rights claims made by citizens or using a citizen logic.

Given that many actors, those that activate citizenship from above and those that mobilize participation from below, shape how different immigrant groups integrate into the local social and political fabric, competing logics about immigrant inclusion may be at play (Greenwood et al., 2009; Jørgensen, 2012). Different rationales can clash or align to motivate the mobilization of resources towards—and advocacy of—urban undocumented residents. In cities with large immigrant populations such as New York and Paris, politicians’ pro-immigrant actions such as protection from deportation or resources earmarked for immigrant issues can influence immigrant voters or voters sympathetic to their concerns. Universalistic missions of government bureaucracies, especially schools, mobilize resources for undocumented residents (Marrow, 2009). Ethnic and non-ethnic community-based organizations advocate for marginalized immigrant residents; religious institutions welcome and sometimes organize undocumented parishioners; and labor unions can vouch for immigrant workers, regardless of legal status. The inclusive missions of these organizations may increase their numbers of immigrant members. Foundations may support the inclusive, democratic values that such organizations embody. Employers can also support certain rights for undocumented residents, especially to protect their workers from deportation; other rights, such as labor organizing and worker rights, receive more ambivalent support.

Due to the influence of these organizations in shaping policy and practice, the process of immigrant incorporation differs not only between states *but* within them as well. The impact of government and civil society organizations on citizenship results from the *interactive* dynamism of these unequal actors, emerging from top-down access and bottom-up mobilization (Penninx et al., 2014). This produces distinct patterns of social, political, and economic incorporation of undocumented immigrants. Community organizations, which advocate for and help realize employment and worker rights, civil and cultural rights, language and religious expression, and access to political participation, are crucial interlocutors (with the state) for politically marginalized groups such as immigrants. Moreover, local ethnic, immigrant, and religious organizations have proven effective in engaging residents, enabling them to represent immigrant concerns to government officials and other powerholders (Castañeda, 2012; Cordero-Guzman, 2005). States in turn shape the financing and structure of local organizations (Bloemraad, 2006). These actors offer undocumented youth access to rights-based services, avenues to participate in social life and develop a sense of belonging, and even access to legal status.

Immigrant incorporation occurs at the meso-level as locally-based actors govern access to social, economic, and political opportunities for residents. The local COFI can magnify or contest national policy; it exerts its own agency on immigrant incorporation. Individuals’ citizenship experiences derive from the characteristics of the COFI and their position relative to it. For analyzing these legally precarious residents, citizenship offers a particularly useful set of modes of incorporation. Four possible avenues of formal and substantive citizenship through which cities might incorporate undocumented residents include offering them opportunities to acquire nationality, access rights, engage with the political system, and/or develop a sense of belonging or identity based in the new context (Bosniak, 2006). First, cities may facilitate the regularization process in which undocumented residents gain a visa by offering them necessary resources, advocating for them, and directly challenging government or agency practices. Next, even without according them formal status, cities give city-based sets of rights to undocumented residents. Despite their lack of legal status, undocumented youth possess the right to elementary and secondary education in both countries. Undocumented students in New York City may study at public and private universities although federal and state financial aid is unavailable. The resulting cost makes college unlikely. Undocumented students in Paris can study in a few free or low-cost post-secondary tracks but do not possess the national identification number needed for most universities’ admission. Thus, in each city they most frequently find themselves in a precarious labor market in which employers pay low wages and abuse undocumented workers. While most undocumented residents cannot participate in formal politics, civil rights, such as the right to protest, allows some to participate in social movements. Lastly, each of these means of inclusion have allowed undocumented youth experiences in a settled community and in public institutions (Conover, 1995), which gives them different degrees of belonging. Attending secondary schools, for example, is one inclusionary practice that shapes undocumented youth in ways like their documented classmates (Gonzales, 2011).

#### The cases of New York and Paris

Scholars evaluate New York as having a vigorous and democratic immigrant incorporation (Hochschild & Mollenkopf, 2009).^2^ In contrast, scholars problematize the integration of immigrants in Paris, often evoking a lack of civic and political representation, suburban riots in which they have been involved, and other indicators of non-incorporation (Bertossi & Duyvendak, 2012; Maxwell, 2010). One central factor for these outcomes is the lack of support that French immigrants receive from ethnic organizations (Castañeda, 2012; Maxwell, 2010), which were limited by law until 1981. A fear of ethnic ‘communitarianism’ follows French public philosophy that expects immigrants to assimilate as individuals into the *République*. We would expect undocumented youth in Paris, like their legal immigrant counterparts, to also receive little support from organizations there. Instead, organizations in Paris have come together to regularize thousands of undocumented youth.

New York City, on the other hand, has developed a city branding campaign around it being a model city of immigrant integration (De Graauw, 2015). Examples include a city-sponsored Immigrant Heritage Month with cultural events, the Mayor’s Office of Immigrant Affairs coordination of language access programs and more, the City Council allocation of $19 million towards DACA outreach, and the city’s municipal identification card program (IDNYC), intended to facilitate inclusion for undocumented residents.^3^ Its immigrant organizations are well-coordinated and wield leverage in the public arena (De Graauw, 2015). Despite the broad popular and public support of immigrants in New York, a fundamental weakness lies in its lack of leverage regularizing its undocumented residents. The young undocumented informants in my study had, despite their lack of legal status, nonetheless developed a stable, adult social life, showing evidence of certain spaces of inclusion outside of formal national citizenship.

In contrast, the Paris young undocumented informants in my study, most of whom had obtained a legal status, nevertheless continued to face daunting obstacles to an integrated adulthood. How to explain this divergence wherein Paris helped undocumented youth gain legal status yet was less effective in integrating these youths while New York provided a setting for integration *without* offering youth legal status? This article argues that the influence of nation-state policies *and* city-level organizational dynamics create these different contexts for undocumented youth.

Despite many similar social goals for immigrant residents in New York City and Paris (e.g. economic incorporation, democratic participation, lack of social unrest), these cities’ organizational fields produce different outcomes for their undocumented youth. This article seeks to explain this empirical puzzle by examining these organizational fields, especially the nature of links between immigrant organizations and local government. This emergent constellation of actors influences immigrant incorporation according to certain institutional logics, resulting in specific kinds of opportunities for outsider groups like undocumented youth. New York City’s more cooperation-oriented organizational field allows a laissez-faire, informal inclusion for undocumented youth. The legitimate citizen-based modes of civil society-government contestation in Paris have created an important niche opening in visa acquisition. Undocumented youth include those who entered a country as a minor without inspection by customs officials, overstayed a temporary visa, or lost a legal residence status (e.g. asylum-seekers). Residing without legal status in France and the United States is an administrative or civil violation, not a criminal one.^4^

## Methods

This project collected data using longitudinal ethnography in Paris and New York with undocumented youth and organizations that support them, including analysis of relevant organizational and legal documents. Given the tendencies of organizations to have policies that are loosely coupled with practice (Thornton et al., 2012), field-level ethnography (Zilber, 2014) supports a rich, practice-based representation of interorganizational dynamics. I define the field examined here as the local organizational field managing undocumented youth. The influence of many actors on this subgroup of immigrant youth coalesce into a relatively institutionalized field, making it possible to identify the positions of state, market, and civil society organizations (Chhotray & Stoker, 2010). Relationships and interactions between actors and within organizations over time enable an accurate representation of field-level dynamics and “rules in use” (Ostrom, 1999, p. 38; Hallett & Ventresca, 2006).^5^

Having worked with immigrant organizations in each setting prior to this project, I selected two immigrant-rich neighborhoods in two best-case scenario cities for undocumented residents. In New York, I began recruiting a dozen male undocumented Mexican-origin youth^6^ from two high schools in 2008. In Paris, I recruited a dozen male undocumented youth, beginning in 2010 with associations that support them. A third of the Paris cohort were Chinese, a third sub-Saharan African, and third mixed between North African, Eurasian, and Brazilian; this represents the ethnic proportions of undocumented youth that I encountered in the two neighborhoods in Paris where I initiated my field work. A theoretical sampling logic pushed me to recruit four Paris and five New York informants through snowballing techniques in order to better represent more socially isolated youth in Paris and more organizationally integrated in New York. Each city cohort, then, includes those who are closely connected to organizations, loosely connected to organizations, and unconnected to organizations. The experiences of these two-dozen male undocumented youth from 2008 until 2016 guided the representation of the institutional field in each city. These representations are based on between 25 and well over 200 interactions (e.g. hang out, talks or observation in a variety of contexts) with each informant. For example, regarding the two young men whom I highlight below, I have interacted with Chen over 50 times and with Ernie over a hundred times.^7^ I used field notes from this participant-observation to evaluate informants’ social lives in a variety of spheres including schooling, family, work, housing, health care, public life, criminality, peer group, romantic relationship, and transnational practices. I noted the organizational contexts in which my informants experienced inclusion or exclusion in these social domains. Working with these youths over the long-term built trust and illuminated the frequent pattern of punctuated or occasional—but important—organizational participation as they became adults.

In addition to participant-observation with these youths and their families as they interacted with organizations, I conducted interviews with and participant-observation in 19 organizations in New York and 18 in Paris. These organizations supported immigrant youth in providing educational-, legal-, social- and family-based services, at both the neighborhood- and city-level of my informants. I then mapped local and national actors and where relevant, their broader organizational categories (e.g. civil society, labor market, government agency), onto the constraints and opportunities available to my informants, an iterative process.

Thus, the representation of the organizational fields (1) begins with the experiences of undocumented youth, (2) is confirmed through data collected directly from the organizations, and (3) triangulated with the media (especially newspaper articles and organizational websites) and social media representations of organizational activity. Regular feedback loops between each of these datasets produced a representation of the most salient internal and external dynamics of organizations. With this basis in practice, this project’s approach avoids the pitfalls associated with organizational policies being decoupled or loosely coupled to practice.

## Comparing city-based organizational fields of immigrant incorporation

Nearly a quarter of Parisians and over a third of New Yorkers are immigrants (see Table 1 below). About a quarter of those immigrants are undocumented in Paris and one in six in New York. Youth, those who arrived under 18 years of age but are under 30, make up about one in nine undocumented in Paris and one in six in New York.

**Table 1:** Selected Populations, Paris and New York

	Paris		New York	
Population	2,234,105	100%	8,185,314	100%
Immigrants	509,376	23%	3,042,315	37%
Undocumented Immigrants	129,604	6%	499,000	6%
Undocumented Youths (15–30 years old with >5 years residence)	15,000	1%	79,000	1%

Sources: Lobo and Salvo (2013), New York Immigration Coalition (2012), INSEE (2011), Secrétariat général (2011); Author estimate

State, civil society, and market actors play different roles in each city. In Paris, the state—embodied in the city’s Prefecture—plays a more central role than in New York. The Prefect both directs police and distributes visas. Civil society, with associations claiming specific sets of rights (e.g. worker, housing, immigrant), maintains a more adversarial stance to make claims on the state. The state limits the institutionalization of ethnic institutions in two ways: by de-legitimizing ethnic organizing and by weakening the connection between employers and ethnic networks through labor law. Rights-based organizations coordinate around a *citoyen* (citizen) logic to make claims on the state, permitting incorporation of undocumented youth.

In New York, the division of powers limits the agency of the local and state governments. Unlike in Paris, the locally-based state cannot offer legal status to undocumented youth. Instead, civil society and city and state government have cooperated to protect undocumented residents from deportation risk, creating a buffer from federal enforcement. Civil society is organized by an ethnic logic (and ethnic voting blocs), and a few organizations coordinate associations’ claims on the state. With looser federal oversight, ethnic communities and employers have an institutionalized relationship. With available low-end jobs, New York offers its undocumented youth a socioeconomic incorporation.

### Paris

Scholars have framed the Minister of the Interior and the National Assembly, on one hand, and *département* prefects on the other, as making and implementing French immigration and integration policy (Hayward & Wright, 2002). While prefects, the lone representatives of the national state at the *département* level, wielded vast executive and administrative powers in the past,^8^ today municipal and regional actors have grown in importance, municipal governments have looked to civil society actors to help implement social programs (Nicholls, 2006). Even with the multiplication of state actors, prefects’ influence remains strong and scholars have downplayed the contributions of civil society to French integration policymaking (Noiriel, 2007; Wihtol de Wenden, 2011). An asymmetrical power balance has skewed negotiations with the state and limited the impact of integration-related social movements.

In June 2004, parents, educators, collectives of undocumented residents, unions, and associations came together in Paris to discuss what to do about the situation of undocumented youth. The mission of the resulting network, the Education Without Borders Network (RESF), was to change the law to allow these populations to grow up in France and to protect them from deportation. In the decade since its birth, RESF has leveraged members’ citizen standing to make human rights claims, shifting the dynamics of Paris’s COFI and facilitating undocumented youth visa acquisition.

COFI actors can provide services and protections, restrict access, or change legal status of undocumented youth. Promoting a policy of non-family, ‘highly-skilled’ migration, the French legislature and Department of the Interior directives have enacted policies that make immigration and subsequent regularization more difficult (Tetu-Delage, 2011; Cette France-là, 2012). Préfectures are primary actors in enforcing these new restrictions, including by doing street identity checks of those suspected of being undocumented. In 2006, a change in the law took away the right to regularization after 10 years of residence and replaced it with a discretionary policy. Then Minister of the Interior Nicolas Sarkozy began a more aggressive deportation policy, including detaining guardians as they waited for children after school in a neighborhood where I conducted my research. President Hollande continued increasing annually the quotas of deportations established under former President Sarkozy (Ministère de l’Intérieur, 2016a).

This process of individuals crossing the legal boundary on a discretionary basis simultaneously reinforces the legal boundary. Police and bureaucratic checks of the national identification card reinforce the divide between French nationals and non-nationals. As Chen, an informant, was in the process of applying for a visa, undocumented Chinese workers were arrested and deported from the nearby train station. Migrants understand that their status has been shifted at the discretion of the state, a disciplining process that does not empower them with newfound rights nor encourage political participation. While temporary visas (“titres de séjours”), most with work permission, have increased by nearly 10% from the period 2008–2010 to 2012–2014, naturalizations in France have dropped by about half in the same time period (Ministère de l’Intérieur, 2016b).

In contrast to this restriction, schools play an inclusionary role in forming citizens out of children that adult immigrants do not experience (Gleeson & Gonzales, 2012). Education for minors is a universal right, and school is the most important public institution for undocumented youth. Teachers have a universal mission to serve all children, which can translate to quite radical claims-making when this universality is applied in other contexts. With the Parents’ Association Federation (FCPE) network structure as a guide, RESF recruited and established school leaders at each school. These leaders act as the school liaison for other RESF action, resources, and information, most importantly neighborhood *permanences* and the listserv. Along with other teachers, school social workers, and staff, these leaders often act as a first contact, someone in a school who serves as a bridge to other resources for undocumented youth. Active teachers tend towards the left of the political spectrum that espouses a more inclusive philosophy towards immigrants, though RESF does not formally align with any political party.

RESF also has extended these initial alliances to link with local representatives, who have embraced new forms of participatory democracy. The density of representation increases with the imbrication of neighborhood, district, city, and regional legislatures in addition to their national level counterparts. RESF then leverages this local strength— connections with schools, associations, and representatives—to counter restrictive national discourse and policies by proving a useful mediator to the Préfecture. Local representatives interact with civil society by 1) providing certificates to undocumented youth and their families in ‘godparenting’ ceremonies, 2) writing letters to support regularization applications, 3) participating in protests, 4) speaking at RESF events, and 5) providing an undocumented youth contract to protect them against deportation. Youth use the resulting documents as proof of social integration when they apply for regularization.

In contrast to civil society organizations that are subcontracted by the government (Nicholls, 2006), RESF’s independence from state financing alters its relationship with state actors. Cooptation by the government has been accomplished by appointing outside leadership to run associations, determining criteria associations should use to carry out their missions, and attaching funding to specific desired actions. Associations with similar rights-claiming missions receive much of their funding from various governmental sources, leaving them more predisposed to respond to governmental concerns. RESF actions require only donated space from unions, social centers, and occasionally, district mayors. With little to gain from action except recognition and cessations from the government, RESF has been able to raise the transaction costs of managing sans-papiers youth. For example, RESF regularly protested, escalating claims-making on the state. Anxious to avoid dealings with a group of potentially problematic students in symbolic public spaces, the Préfecture has agreed to meetings with RESF leaders and youth. Similar action has also swayed courts where the Préfecture threatens deportation. RESF activists use various methods to oppose police round-ups.

Along with a few human rights organizations, RESF has become an efficient means of building and transferring specialized information on undocumented residents and residence, including information on the social position of *sans-papiers*, their means of entry, problematic social situations, where they live, and how to communicate with them. RESF members have regularly accompanied undocumented youth in administrative appointments to apply for regularization. RESF has regularly entered into negotiations with the Prefect to regularize groups of undocumented youth. This practice follows the Préfecture’s policy of associationalism (Nicholls, 2006) in which the state makes grassroots civil society partners partly responsible for social problems. These practices institutionalize RESF in the organizational field, insofar as the Prefect views them as a legitimate and useful (and sometimes vexing) interlocutor. Without RESF, these youths would not gain visas.

RESF has activated several legitimate discourses in its transition from outsider social movement to an outside collaborator with the Préfecture. The network leverages the universal legitimacy of schoolchildren to build political alliances with local government and civil society. Its confrontational tactics are highly legitimate, given France’s historical democratic performances. Finally, the Prefecture’s social goals and desire for public order justify the interaction with RESF. Through its strategic linkages in governance and exploitation of legitimate institutional logics, RESF coordinates an impressive mobilization for undocumented youth who are eventually regularized. In this arena of claims making, undocumented adults do not benefit from the same base of universal ethics of participating in public institutions, like children in schools. A Prefecture clerk told me that without French fluency, long-term residents would not qualify for a visa.

France’s statist logic creates ‘governance with government’ (Elgie, Grossman, & Mazur, 2017), with the state the central actor. At the intersection of civil society and the state, RESF coordinates local opposition to the Prefecture, which reduces undocumented youths’ deportation risk and allows many to regularize their status. Open contestation (legitimized by a citizen logic) and eventual negotiation (driven by the state’s associationalist logic) characterizes the interactions between the state and these civil society and local government actors. The logic of the state-influenced market economy, however, limits market actors, including flexible workforce policies (ibid.). National inspectors raise the cost of using unauthorized workers, and employers are hesitant to hire them in this environment. Chen, for example, told me he could do at-home piecemeal sewing but Chinese street businesses would not hire him without a visa. Work opportunities are limited for young people in general, making it particularly hard for undocumented or newly documented immigrant youth to find steady work. Chen stayed working longer than other informants at his first job after being regularized, but the part-time work and lack of better possibilities limited his socioeconomic integration.

### New York

Multi-level governance strongly affects New York’s COFI. Historically, the federal government has claimed full powers over immigration while leaving integration policy to states and localities. The boundaries of the federal-local division of powers have shifted over the past two decades, with more initiative taken by localities and a stronger federal-local link than in the past (Cortez, 2008; Varsanyi, 2010).

Local government actors in New York have shifted policies and practices to buffer federal oversight of undocumented residents. Former Mayor Bloomberg implemented a “Don’t Ask, Don’t Act” policy on immigration status for all city employees. By encouraging interactions between undocumented residents and police, fire, and other municipal employees, this policy intended to facilitate maintenance of public order. When Ernie, an informant, sees police at his neighborhood park, he views them skeptically but does not worry about deportation. With the same rationale, the city has passed a policy of non-cooperation with the federal Immigration & Customs Enforcement agency (ICE). Unless charged with specific ‘serious’ felonies, local police do not cross-check undocumented residents’ data with federal databases when arrested, preventing possible deportations. In a context of sharply increased deportation policy under Presidents Bush and Obama, the impact of these policies should not be understated.

In addition to these protections from deportation, New York has expanded its integration programs. Intended to promote and coordinate immigrant organizations and businesses in interactions with the city, the Mayor’s Office of Immigrant Affairs has grown in scope and is now considered a national leader in immigrant inclusion. Two initiatives seek to legitimize undocumented youth through identification. The City Council allocated $19 million to immigrant organizations towards outreach for the DACA program. In 2015, the City Council passed a municipal ID card program to ease undocumented residents’ lack of US-based formal documents. A liberal language policy offers city documents in the top six languages of immigrants (De Graauw, 2015). Finally, though the $78 billion budget for fiscal year 2016 in New York City (New York City Office of Management and Budget, 2016) does not earmark money for immigrant services, budget-line categories such as community development, youth services, cultural affairs, education, and many others go to ethnic organizations and other organizations that serve immigrants. This public funding supports private organizations’ ethnic events, including a few Mexican cultural events that Ernie has attended.

What encourages mayors and city legislators to support these pro-undocumented resident programs? Several imbricating logics are at work. First, New York’s government has taken several steps to develop its legislative representation of immigrant residents, following its history of ethnic politics. Attempts at ethnic recognition and representation are at the core of these steps. New York’s democratizing move in 1989 expanded its number of city council representatives from 35 to 51. Redistricting in 1991, 2002, and 2012 then draws district lines around minority populations to encourage minority representation (Mollenkopf, 2013).

A second important dynamic that underlies these pro-immigrant policies is the role of community organizations in elections. With machine politics, politicians often fund community-based ethnic organizations who then use their embeddedness in communities to deliver votes (Marwell, 2004). New York’s many nonprofits, community-based organizations, religious, labor unions, and other organizations play a vital role in articulating immigrant needs, providing social and legal services, and advocating for immigrant issues (Cordero-Guzman, 2005; De Graauw, 2015). With New York’s historical legacy of immigration and ethnic incorporation (Foner, 2000), ethnic-based organizing is widespread and legitimate. Influenced by discourses of the discrimination of ethnoracial minority groups, most powerholders frame the exclusion of undocumented youth as a racialized civil rights issue. For this reason, ethnic organizations are highly legitimate as representatives of residents’ concerns (Hum, 2014). Articulating community needs has not been enough to pressure legislators, however; organizations exploit politicians’ desire to access voting citizens at events and media coverage (De Graauw, 2015). The New York Immigration Coalition, an umbrella organization of nearly 200 organizations, channels messaging and coordination with politicians.

Similar to this inclusion provided by ethnic organizations, schools offered rights and a context for belonging (i.e. substantive citizenship); they have not been a setting for mobilization, however. Furthermore, this inclusion terminates with high school. At Ernie’s high school, teachers and guidance counselors did not know about options for undocumented students and did not offer advice for post-secondary plans. The City University of New York (CUNY), a large multi-campus public university with a large immigrant student body, has targeted outreach to increase admission of Mexican students, a group with a high percentage of undocumented residents (Smith, 2013). CUNY Citizenship Now offers free workshops on how to naturalize for those eligible.

Legal exclusion thus coalesces for undocumented youth around the challenges of paying for college without financial aid and finding a job outside of federal regulations. In contrast, DACA allows most undocumented youth to find work in regulated sectors. Government and civil society actors could support recruitment of DACAmented New Yorkers to CUNY. Financial aid for college would mitigate this exclusion but popular support in New York City or State has not been sufficient for legislation; some private sources now offer selective scholarships to undocumented students.

While many undocumented youths pointedly feel the exclusion from obstructed access to college, other city actors value their presence. With partisan politics largely an afterthought in New York, organizational-level logics also strengthen this government-civil society consensus. Unions and religious organizations see potential members; employers desire large numbers of low-wage accommodating workers; progressives value their potential inclusion; libertarians like the lack of federal interference. While this portrait of support seems to offer many potential strategic openings, political support requires momentum. Coordinating the above interest groups would require benefits to outweigh costs for all actors and long-term benefits must remain clear (Nicholls, 2005).

Local state and civil society cooperation has buffered the influence of the federal oversight, creating space for employers of undocumented residents. Employers make use of ethnic networks to recruit workers, and sometimes prefer accommodating undocumented workers (Saucedo, 2006). Given more choice in the labor market than in college, undocumented youth most often resort to working full-time. Ernie, for example, has used his family’s networks and a false social security card to find full-time jobs in three factories since high school graduation. The high cost and unclear payoffs of college attendance have deterred Ernie from continuing his education.

## Micro-level citizenship experiences

This article’s focus is on meso-level dynamics. As a starting point for understanding the impact of different organizational fields, here I introduce vignettes of two typical undocumented young men.^9^ Though both had been undocumented when I began working with them, Chen gained a renewable visa. In contrast, Ernie did not gain legal status but has leveraged local resources to build a family.

### Paris, January 2016

At a local Prefecture office a few weeks earlier, Chen, 23 years old and formerly undocumented, had successfully renewed the visa that I, with the support of two organizations, had helped him to obtain 4 years earlier. We had planned to meet up this winter afternoon and head out from the converted one-bedroom apartment he shares with his two parents and France-born younger sister in a Chinese enclave in Paris. “I’m still working in the same kitchen,” he grumbled in French, “I’m not full-time, I get the SMIC [monthly minimum wage in France]”. He vowed his vocational high school degree in heating/cooling was to help him get papers only; his dream was to open a store with his dad. Next year he would apply for a long-term 10-year visa. Although French sometimes tripped him up, Chen’s facial vocabulary compensated. “Our store will not be like that,” he disapprovingly nodded at a new boutique, “it will be more Chinese”.

### New York City, June 2016

Ernie and I sat on the lower level of the bunk bed, his younger US-born brother playing video games next to us. Ernie, 25 years old and undocumented, asked me in Spanish about applying again for DACA privileges; he had not re-applied after his initial temporary period expired. “I still have the same job,” he complained, indicating that the program benefits had been limited for him; he has held full-time factory jobs since he graduated from high school in 2009. I had volunteered with one nearby Latino organization to help with DACA applications in 2012, and suggested he go there to learn more. Over lunch, his mother and he asked how to get paid for their overtime hours. A nearby organization had successfully fought for other undocumented workers’ rights although discrepancies between work and office schedules made it difficult for them to connect. Despite this sense of unrealizable worker rights and rising rent pressure, Ernie said he was glad his 5-year-old son was growing up in New York. “He is a New Yorker!” he stated firmly. “Aren’t you, too?” I quizzed in Spanish. “Yeah, I guess so,” he said in English.

These vignettes challenge the myth that people can be categorized as either citizens with full rights or non-citizens without rights (Cohen, 2009). Though on track to become a citizen, Chen’s integration remained more limited. Having national citizenship is an important but not all-encompassing facet of citizenship. Undocumented residents possess different sets of rights depending not only on the nation-state but also the characteristics of the local organizational field. Ernie’s experiences of citizenship—from presence, rights, and participation—in everyday life strengthened his sense of belonging.

Paris incorporation is more on the socio-political axis than the socio-economic one. By 2016, one Paris informant had returned to Brazil, eight had gained visas (six through RESF) including one who became a citizen, and three remained without visa. The process of applying for a visa necessitated youth to participate socially in new ways, including bureaucratic experiences. My informants faced a particular and exaggerated version of the labor market woes faced by French youths and immigrants in general. Chen, for example, expects to gain a 10-year visa but his vision of work opportunities remains limited. Such difficulties in the labor market give undocumented (or recently documented) youth a sense of exclusion, unable to provide reliable wages to their needy households or even to facilitate romantic relationships. Chen’s Chinese background does not connect to French discourse or civil society the way that being a Mexican or Latino immigrant in New York gives Ernie a foothold into an American identity and civil society. Thus, even after gaining legal status and its accompanying social rights, youth in Paris fail to develop a well-rooted sense of belonging.

By 2016, one New York informant had moved back to Mexico, one had gained a green card using a limited loophole, community organizations helped seven to successfully apply for DACA (one did not renew), and three did not apply. Without the benefits of formal citizenship, New York youth experience a socioeconomic incorporation albeit one in a subordinate position. Like Ernie, most of my New York informants work for low wages in no-growth but full-time jobs in restaurant kitchens, delis, construction, and factories. This outcome stems from the looser labor market and job entry facilitated by strong ethnic networks. Exceptional are two informants with college experience who have leveraged DACA benefits to work in a community organization and a research lab; they also are the only ones who have participated in the Dreamer movement. Undocumented and DACAmented youth felt symbolically included and contributed to society through work and family, they sensed their socioeconomic marginalization as they see their peers more smoothly attained postsecondary experience and better jobs with less exploitation. The buffer from federal restriction let youth experience everyday inclusion, from using New York’s public transportation and visiting Times Square to joining sport leagues. This opening and the availability of stable low-paying jobs, however, provided necessary resources and built a sense of belonging in undocumented male youth and their working-poor families.

## Discussion

Local governance shapes immigrants’ citizenship possibilities (Dekker et al., 2015; Jørgensen, 2012; Smith & McQuarrie, 2012; Penninx et al., 2014). This article set out to understand how the meso-level organizational fields managing undocumented youth shape citizenship experiences for such young people. The COFI framework, focusing on governance constellations and institutional logics, allows a dynamic representation of unequal actors and their schema for undocumented youth issues. Urban undocumented youth can experience citizenship in diverse ways, formal and informal, in acts and in sense of belonging.

Findings, based on multilevel longitudinal ethnographic data, showed surprising socio-political outcomes in Paris and New York. Cities articulate some similar goals of immigrant incorporation but end with different results given different sets of actors working with different institutional logics. Immigrants surely represent themselves in both organizations and government to a greater extent in New York than Paris. The fragmented division of powers, strategic alliances, and contentious citizen-based claims, however, favor the legalization chances of undocumented youth in Paris. Despite political underrepresentation in each city, organizations use logics of democratic legitimacy, public order, and market efficiency to institutionalize social movements into governance actors. The strategic opening exploited by Paris civil society actor RESF responds to a discourse of assimilative citizenship (Nicholls, 2013) rather than that of the ethnic/immigrant worker of New York.

When interpreting the effects of the COFI on undocumented youth, we can see nuance in incorporation outcomes. The local actors in Paris and New York filter France’s civic assimilative model and the US pluralist model, following much more contextualized logics than we would expect of either of these models. For undocumented youths, socio-political inclusion is easier to achieve in Paris than New York. In Paris, social movement tactics combine with local social policy to gain a foothold for undocumented youth. In New York, Civil Rights Era-influenced ethnic politics convince organized interests to support them.

Studies have shown how local governance actors shape cultural integration (e.g., Penninx et al., 2014), political integration (e.g., Varsanyi, 2010) and civic/associational integration (e.g., Castañeda, 2012; Smith, 2013) of urban immigrants. This study extends the fruits of such an approach by identifying how local governance actors specifically shape incorporation of undocumented youth. If and when civil society shaped the transaction costs of managing undocumented youth, its power vis-à-vis government increases. Greater contestation of policy in Paris afforded RESF logics access to formal citizenship while greater cooperation with local government in New York afforded greater participation and sense of belonging.

Rather than focusing on policy alone, this article shows how the practices of—and between—government, civil society, and market actors in the COFI shape uneven lived citizenship experiences of undocumented youth as they come of age. The formal right to reside, more easily attained in Paris, does not necessarily equate with participation or a sense of belonging. Marginalization in New York did not preclude participation or a sense of belonging. Although undocumented residents are often portrayed as outside the system—“un-citizens” in the words of one scholar (Nash, 2009), this locally-based empirical study casts them instead as marginalized citizens.

## Footnotes

1 Deferred Action for Childhood Arrivals (DACA) was an executive action under President Obama that offered temporary, renewable relief from deportation and work permits to qualified undocumented youth. For more background, please see (Gonzales, Terriquez, & Ruszczyk, 2014).

2 These authors assess immigrant political representation as stronger in New York City than in Paris.

3 By offering an identification card to all residents, including undocumented ones, IDNYC was intended to expand participation and limit the effects of immigrant ‘illegality.’ For more information on such programs, see De Graauw (2014).

4 In each country, the use of deportations to punish such violations has sharply escalated beginning in the mid-2000s (Gonzalez-Barrera & Krogstad, 2014; Ministère de l’Intérieur, 2016a).

5 The cross-case comparison between Paris and New York fields sensitized me to variation in these dynamics, enriching the data collection and analysis. Fieldwork and revisit work seesawed between the settings and created an analytical dialogue between the cases. This ethnographic practice of juxtaposing the experiences of youth between cities as well as between organizational fields illuminated aspects of each case.

6 The selection of male primary informants facilitated an intimate ethnography with informants who were, at the start of the study, teenage youths.

7 Ernie and Chen are pseudonyms.

8 Although the French state has been characterized by the highly centralized model of prefects leading its 100 *départements*, decentralization policies have been implemented beginning in the 1980s. *Départements* are the administrative level between the region and the county, and Paris is its own *département*.

9 A full presentation of the lived experiences of other undocumented youths is beyond the scope of this article. More information can be found at (Ruszczyk, 2015). For more information on gendered aspects of undocumented youth experiences, please see (Ruszczyk, in press).
